# *Plasmodium falciparum* alters the trophoblastic barrier and stroma villi organization of human placental villi explants

**DOI:** 10.1186/s12936-024-04960-9

**Published:** 2024-05-01

**Authors:** Carolina López-Guzmán, Ana María García, Juan Diego Ramirez, Trinidad Torres Aliaga, Alejandro Fernández-Moya, Ulrike Kemmerling, Ana María Vásquez

**Affiliations:** 1 Grupo Malaria, Facultad de Medicina, Universidad de Antioquia, Calle 62 #52-59 Torre 1, Laboratorio 610, Medellín, Colombia; 2 Escuela de Microbiología, Universidad de Antioquia, Calle 67 # 53-108, Bloque 5, Oficina 5-135, Medellín, Colombia; 3 Instituto de Ciencias Biomédicas, Facultad de Medicina, Universidad de Chile, Independencia 1027, Santiago, Chile

**Keywords:** Malaria, *Plasmodium falciparum*, Placenta, Histopathology, Pathology (source: MeSH NLM)

## Abstract

**Background:**

The sequestration of *Plasmodium falciparum* infected erythrocytes in the placenta, and the resulting inflammatory response affects maternal and child health. Despite existing information, little is known about the direct impact of *P. falciparum* on the placental barrier formed by trophoblast and villous stroma. This study aimed to assess placental tissue damage caused by *P. falciparum* in human placental explants (HPEs).

**Methods:**

HPEs from chorionic villi obtained of human term placentas (n = 9) from normal pregnancies were exposed to *P. falciparum*-infected erythrocytes (IE) for 24 h. HPEs were embedded in paraffin blocks and used to study tissue damage through histopathological and histochemical analysis and apoptosis using TUNEL staining. Culture supernatants were collected to measure cytokine and angiogenic factors and to determine LDH activity as a marker of cytotoxicity. A subset of archived human term placenta paraffin-embedded blocks from pregnant women with malaria were used to confirm ex vivo findings.

**Results:**

*Plasmodium falciparum-*IE significantly damages the trophoblast layer and the villous stroma of the chorionic villi. The increased LDH activity and pathological findings such as syncytial knots, fibrin deposits, infarction, trophoblast detachment, and collagen disorganization supported these findings. The specific damage to the trophoblast and the thickening of the subjacent basal lamina were more pronounced in the ex vivo infection. In contrast, apoptosis was higher in the in vivo infection. This disparity could be attributed to the duration of exposure to the infection, which significantly varied between individuals naturally exposed over time and the 24-h exposure in the ex vivo HPE model.

**Conclusion:**

Exposure to *P. falciparum*-IE induces a detachment of the syncytiotrophoblast, disorganization of the stroma villi, and an increase in apoptosis, alterations that may be associated with adverse results such as intrauterine growth restriction and low birth weight.

**Supplementary Information:**

The online version contains supplementary material available at 10.1186/s12936-024-04960-9.

## Background

The most vulnerable population to *P. falciparum* infection includes pregnant women and children under 5 years old. Specifically, during pregnancy, *P. falciparum* causes approximately 150,000 fetal deaths and 10,000 maternal deaths annually in malaria-endemic areas [1]. During *P. falciparum* infections, the infected erythrocytes (IE) can accumulate in the placenta as a result of the binding of the Chondroitin Sulfate A (CSA) receptor present on the Syncytiotrophoblast (STB) and the parasitic antigen VAR2CSA expressed by infected erythrocytes circulating in the intervillous space [2]. The sequestration of infected erythrocytes in the placental villi has been considered the primary cause of adverse effects during pregnancy and histological alterations in the placenta [3–5]. Adverse pregnancy outcomes caused by parasites include maternal anaemia, low birth weight, and intrauterine growth restriction, among others [1, 6, 7]. The presence of IE in the intervillous space of the placenta results in an increase in mononuclear and polymorphonuclear cells that infiltrate the tissue to control the local infection [8, 9]. Both sequestration and inflammation are two crucial factors causing disruptions in the exchange of gases and nutrients between the mother and fetus, as well as disturbances in angiogenic and endocrine mediators. These factors have been linked to low birth weight and intrauterine growth restriction [10, 11].

The human placenta is a temporary organ that plays a crucial role in the successful development of the fetus [12]. It is formed by maternal tissue, which corresponds to the decidua, and fetal tissue, formed by the anchored and free-floating chorionic villi. Free-floating chorionic villi are surrounded by maternal blood in the intervillous space and thus in direct contact with microorganisms present in the blood. The chorionic villi are the morpho-functional unit of the placenta where the placental barrier is located. The placental barrier is composed of the trophoblast, an epithelium formed by a proliferative basal layer (cytotrophoblast, CTB), and a superficial syncytium formed by the STB, basal membrane, villous stroma (the fetal connective tissue that contains the fetal capillary also surrounded by a basal membrane [13]. The STB has critical functions within the placental barrier, including the exchange of gases and nutrients, hormone production, immune tolerance toward the semi-allogeneic fetus, acting as a physical barrier against pathogenic microorganisms, and contributing to the control of infections at the maternal–fetal interface [14].

Despite the existing information about the unfavorable pregnancy outcomes associated with placental malaria caused by *P. falciparum*, little is known about the direct effect of the parasite on the placental barrier. Furthermore, it is essential to assess the impact of infection on STB function, including the production of inflammatory, angiogenic, and endocrine mediators which are crucial for fetal development. Thus, HPEs constitute an excellent alternative to studying the effect of *P. falciparum* infection on the placental structure and function. HPEs have been used to study diverse placental pathologies and other parasite infections [15–17].

HPEs from chorionic villi were used to study the impact of *P. falciparum* on placental tissue integrity and function; this experimental approach helped provide information regarding the first interaction between the placenta and the parasite in a short period, considering the lack of a model to study human placental malaria.

## Methods

### Collection of placental samples and culture of human placental explants

Nine human placentas at term (> 37 weeks of gestation) were obtained from healthy pregnant women delivered by cesarean sections. Exclusion criteria included: pregnancies with specific medical conditions such as pre-eclampsia, diabetes, intrauterine infection, and any other maternal disease. Pregnant women were admitted to the Obstetrics Unit of Clinica Rosario, Medellín-Colombia. This study was approved by the Ethics Committee of the Faculty of Medicine, University of Antioquia (Min No. 015 24/09/2020). Written informed consent was obtained from all the women involved in the study. After delivery, placentas were obtained under sterile conditions and processed within 1 to 3 h. Maternal and fetal surfaces were discarded, and villi tissue was obtained from central cotyledons, by taking approximately five randomly selected 2 × 2 cm fragments of cotyledons from the maternal side of each placenta. These fragments were washed to remove maternal blood, and smaller villi dissections (about 0.5 cm^3^) were obtained. Three chorionic villi explants (average of 100 mg total) were cultured in 2 mL of DMEM/F-12-Ham medium supplemented with 10% fetal bovine serum, penicillin (100 U/mL), and streptomycin (100 μg/mL), in 6-well plates. The cultures were incubated for 48 h before treatment.

### Culture of *P. falciparum-*IE adherent to CSA (FCB1CSA)

The FCB1CSA strain was cultured in A + erythrocytes in RPMI-1640 medium supplemented with 25 mM HEPES, 21.6 mM NaHCO_3_, gentamicin (16 µg/L), hypoxanthine (0.2 mM) (Sigma-Aldrich, Ref. R5886), and 10% A + human serum. Continuous culture was maintained in a haematocrit (Hto) of 5% and a gas mixture of 5% CO2, 5% O2, and 90% N_2_ at 37 °C [8, 18]. Parasitaemia was monitored daily, and the medium was changed every 24 h. Healthy erythrocytes were added thrice a week until a 5–7% parasitaemia level was achieved [8]. Parasites adherent to CSA (FCB1CSA strain) were selected every four weeks to maintain the parasite's phenotype. Briefly, the culture flask was coated with soluble CSA for 2 h at 37 °C with agitation. After a series of PBS 1X washes and the application of a blocking solution, the parasite suspension was added to CSA-coated flasks and incubated for 2 h at 37 °C on a shaker. Subsequently, the medium was aspirated, and a gentle wash with 5 mL of incomplete RPMI 1640 eliminated non-parasitized and non-adherent erythrocytes. Following this, 5 mL of complete RPMI 1640 culture medium (10% human serum and 3.5% sodium bicarbonate) and non-infected erythrocytes at a haematocrit of 2.5% were added. Finally, the flasks were incubated at 37 °C with a gas mixture suitable for *P. falciparum-*IE until the following day. The next day, the culture was transferred to new bottles, and cultivation continued until the parasites were ready for the repeat selection procedure [8].

### Concentration of mature forms of *P. falciparum-*IE in gelatin

Mature forms of *P. falciparum-*IE (trophozoites and schizonts) were concentrated using the gelatin flotation protocol. The *P. falciparum-*IE cultures were centrifuged at 2500 rpm for 5 min in conical tubes. The culture supernatant was removed, and 1% porcine gelatin solution (Sigma) in incomplete RPMI-1640 was added to the *P-falciparum-*IE pellet (1 volume of IE pellet/10 volumes of gelatin). The mixture was incubated at 37 °C for 40 min, allowing separation into two phases. The upper phase, containing the mature stages, was then transferred to a new 15 mL conical tube, and washed twice with incomplete RPMI-1640 by centrifugation at 2500 rpm for 5 min. The parasitaemia of the pellet was estimated by counting *P. falciparum-*IE in a thin blood smear [18].

### Co-culture of HPEs and *P. falciparum*-infected erythrocytes

All placental explants were washed three times with warm PBS 1X before treatment. For each placenta, three groups or conditions were tested: (i) HPEs alone (Control), (ii) HPEs with non-infected erythrocytes (nIE), and (iii) HPEs with *P. falciparum*-IE. Infected erythrocytes with mature stages of *P. falciparum-*IE were added at 10% parasitaemia and 3% haematocrit (corresponding to 30,000 parasites/uL; this parasite density was calculated from the parasitaemia value assuming that 1 µL of blood contains 5 × 10^6^ erythrocytes at 50% haematocrit) [19]. Parasites were suspended in the HPEs culture medium and incubated for 24 h, at 37 °C with HPEs culture gas mix (21% O_2_, 5% CO_2_). After this period, supernatants were collected for viability measurements and functional assessment. A portion of the tissue was stored in Trizol for gene expression analysis via RNA extraction, whereas another part was stored in 10% neutral formalin at room temperature for HPE histological staining and structural analysis. Each assay was performed with at least three placentas from different donors and evaluated in duplicate. A summary of the sample size for each experiment is provided in Additional file 1: Table S1, including characteristics of 9 placentas used for HPE culture and their usage across the experiments.

### Measurement of viability through lactate dehydrogenase (LDH) activity detection

Lactate dehydrogenase (LDH) is an enzyme located in the cytosol, and it is released to culture medium upon cell damage or lysis. Therefore, LDH activity in culture supernatant can be used as a surrogate of cell membrane integrity and thus reflects cytotoxicity. LDH activity was measured using the commercial kit from Roche Diagnostics, "Cytotoxicity Detection Kit," following the manufacturer's recommendations. The evaluation involved a two-step reaction. At first, LDH present in the supernatant of cultures reduces NAD+ to NADH plus H+ by oxidation of lactate to pyruvate. Second, diaphorases catalyze the reduction of tetrazolium salt (INT) to formazan.

After 24 h of HPEs culture, 100 µL of the supernatant was transferred to a 96-well plate in triplicate. Then, 100 µL of the reaction mixture (catalyst and dye solution) from the kit was added to each well. The plate was incubated in darkness at room temperature for 30 min. After that, 50 µL of 2N sulfuric acid (R&D Systems) was added to stop the reaction. The optical density (OD) was measured at 450 nm using a microplate reader (Multiskan™ FC Microplate Photometer, Thermo Scientific™ Massachusetts, United States). Triton X-lysed tissue was used as a positive control to validate the technique, representing 100% LDH release (Additional file 2).

### Quantification of endocrine mediators and angiogenic factors in HPEs supernatant

Concentrations of the hormone human gonadotropin chorionic (βhCG) in culture supernatants of HPEs were determined by enzyme-linked immunosorbent assay (ELISA), using “DuoSet® ELISA kits” (R&D Systems) (Cat. Number: DY9034-05) according to the manufacturer’s instructions. Levels of the angiogenic factors: Placental Growth Factor (PlGF) (R&D Systems) (Cat. Number: DY264), Vascular Endothelial Growth Factor (VEGF) (R&D Systems) (Cat. Number: DY293B-05), VEGF receptor (VEGF R1/Flt-1) (R&D Systems) (Cat. Numbers: DY321B), and Endoglin/CD105 (END) (R&D Systems) (Cat. Number: DY1097) were also evaluated. Samples were added without further dilution. The absorbances were read at the Multiskan™ FC Microplate Photometer at 450 nm and the concentration of βhCG and angiogenic factors were determined in pg/mL by extrapolating the data from the absorbance against a standard curve, normalized for every 100 µg of tissue. The assay sensitivity limits of the kits are: 7.81 pg/mL to βhCG, 31.3 pg/mL to PIGF, 31.3 pg/mL to VEGF, 125 pg/mL to VEGF R1/Flt-1 and 46.27 pg/mL to END.

### Measurement of cytokines by flow cytometry

Cytokine profiles were assessed using the BD Bioscience Human TH1/TH2/TH17 Cytometric Bead Array (CBA) kit (Cat. Number: 560484), which included IL-2, IL-4, IL-6, IL-10, TNF, IFN-γ, and IL-17A. The standard curve preparation, working mixtures, and cytokine reagents were prepared following the manufacturer's recommendations. Briefly, the standard, negative control, and samples were incubated overnight at 4 °C with PE-labelled TH1/TH2/TH17 detection reagent. The following day, a washing step was performed, and the plate was centrifuged to remove the supernatant. A wash buffer was used to resuspend the samples from the wells, which were then transferred to Falcon cytometry tubes for analysis. Finally, the samples were analysed using a flow cytometer (Beckman Coulter Cytoflex), and the data were processed with FlowJo v10.8.1 software. The assay sensitivity limits of the kits are: 2.6 pg/mL to IL-2, 4.9 pg/mL to IL-4, 2.4 pg/mL to IL-6, 4.5 pg/mL to IL-10, 3.8 to TNF, 3.7 pg/mL to IFN-γ and 18.9 pg/mL IL-17A.

### Histological and histochemical techniques

Placental explants were fixed in 10% formaldehyde in 0.1 M phosphate buffer (pH 7.3) for 24 h, dehydrated in alcohol, clarified in xylene, embedded in paraffin, and sectioned at 5 μm. Paraffin histological sections were stained with hematoxylin–eosin (H&E) for routine histological analysis to evaluate the integrity of HPEs and quantify the presence of syncytial knot, fibrin deposits, and infarction. The frequency of villi with alterations was determined based on the total number of villi evaluated in ten randomly selected microscopic fields. Collagen organization in HPEs stroma was assessed using Masson's Trichrome (TM) for qualitative analysis. Sirius Red Picric (PSR) staining was also used for semi-quantitative analysis of collagen organization (Type I and III collagen). Histopathological damage score in H&E sections and collagen histochemistry with PSR were evaluated according to established histopathological scoring principles for research, as previously described [20, 21]; numerical values were assigned to the presence or absence of the event and presented in Table 1, adapted from [17].

**Table 1:** Scores for the analysis of histopathological damage, organization of collagen types I and III and basement membrane thickening

Score	Histopathological damage	Organization of type I and III collagen	Basement membrane thickening
1	Trophoblast/Fetal connective tissue intact	Absence of collagen birefringence	Absence of thickened regions
2	Minor detachment of the trophoblast and/or disorganization of fetal connective tissue	Low collagen birefringence	Between 1 and 2 regions of thickening per villi
3	Almost complete detachment of trophoblast and/or disorganization of fetal connective tissue	Moderate collagen birefringence	Between 3 and 4 regions of thickening per villi
4	Complete detachment of the trophoblast/disorganization or destruction of fetal connective tissue	Strong collagen birefringence	More than 5 regions of thickening per villi

Adapted from [17]

Basement membrane integrity was examined with Periodic Acid-Schiff (PAS) staining for semi-quantitative analysis, focusing on areas with basement membrane thickening and identifying aldehyde groups in proteoglycans within the trophoblast basal lamina and fetal blood vessels in the villous stroma. The total number of villi present in 5 random fields was evaluated. The number of regions exhibiting thickening of the basal lamina in each placental villus was quantified, considering the assigned score (Table 1). Data are presented as the average of regions with villus thickening.

The parameters evaluated in the histochemical histological and histochemical techniques analyses are described in detail in a previous study [21]. The slides were examined by two trained microscopists who conducted histological analysis at different times using light microscopy (Motic BA 310) at 40× magnification (total magnification = 400) with H&E, TM, and PSR and with PAS at 20× magnification (total magnification = 200). The photos were taken with the Motic Images Plus 2.0 Program.

### Immunohistochemistry

Tissue samples were deparaffinated with alcohol and xylene, and antigen retrieval was achieved by steaming the samples in sodium citrate buffer for 30 min. A polyclonal anti-CK-7 IgG antibody (Cat. Number: MA1-06316) was added to assess the integrity of the trophoblast, followed by a secondary peroxidase-conjugated antibody. The antigen–antibody complex was visualized using DAB chromogen, and nuclear contrast was provided by Mayer's hematoxylin. A negative control was established using phosphate buffer instead of the primary antibody. Ten random fields from each sample were selected to determine the frequencies of the studied variables, including trophoblast detachment, trophoblast rupture, and villi denudation. Following the methodology described previously [21]. The slides were examined by two trained microscopists who conducted histological analysis at different times using light microscopy (Motic BA 310) at 40× magnification (total magnification = 400), and the photos were taken with the Motic Images Plus 2.0 Program.

### Apoptosis assessment using the TUNEL assay

The DeadEnd™ Fluorometric TUNEL System (Promega®) (Cat. Number: G3250) was used to measure the number of apoptotic cells in HPEs samples. This system detects fragmented DNA in apoptotic cells by catalytically incorporating fluorescein-12-dUTP at the DNA ends using the recombinant terminal deoxynucleotidyl transferase (rTdT) enzyme [22]. Samples were prepared by cutting 3-micron sections of tissue from paraffin blocks, followed by formaldehyde treatment, and staining according to the manufacturer's recommendations (Promega). Green fluorescence was employed to identify apoptotic cells, while DAPI staining served as a blue background. A positive apoptosis control was utilized, involving HPEs treated with TNF-α for 24 h.

### Collection of placentas naturally exposed to *P. falciparum-*IE for the validation of ex vivo results

To confirm and complement the results observed in HPEs exposed to *P. falciparum-*IE ex vivo, placentas from pregnant women with malaria by *P. falciparum* were included. Paraffin-embedded tissues were obtained from pregnant women who had previously participated in a study conducted in the Urabá-Antioquia region during 2005–2007. The selection criteria for these tissues included and analysed in this study was the availability of samples in paraffin blocks. The selected tissues, which were used to validate the ex vivo analyses in this study, were categorized into three groups, each with a sample size (n) of 6, based on both sample availability and infection status: Group 1 (Control) consisted of women without malaria, Group 2 women with a history of past placental malaria, and Group 3 women with active placental malaria.

### Statistics

All experiments were conducted using at least 3 placentas from different donor. Experimental data were presented as mean (ME) ± standard error of the mean (SEM). A one-way repeated measures ANOVA test was employed to compare between the control and experimental groups, followed by the Tukey test for multiple comparisons. Values with p ≤ 0.05 were considered statistically significant. Figures and statistical analyses were performed using GraphPad Prism version 10.

## Results

### *Plasmodium falciparum-*IE increases cell damage in HPEs exposed ex vivo

**Fig. 1:**
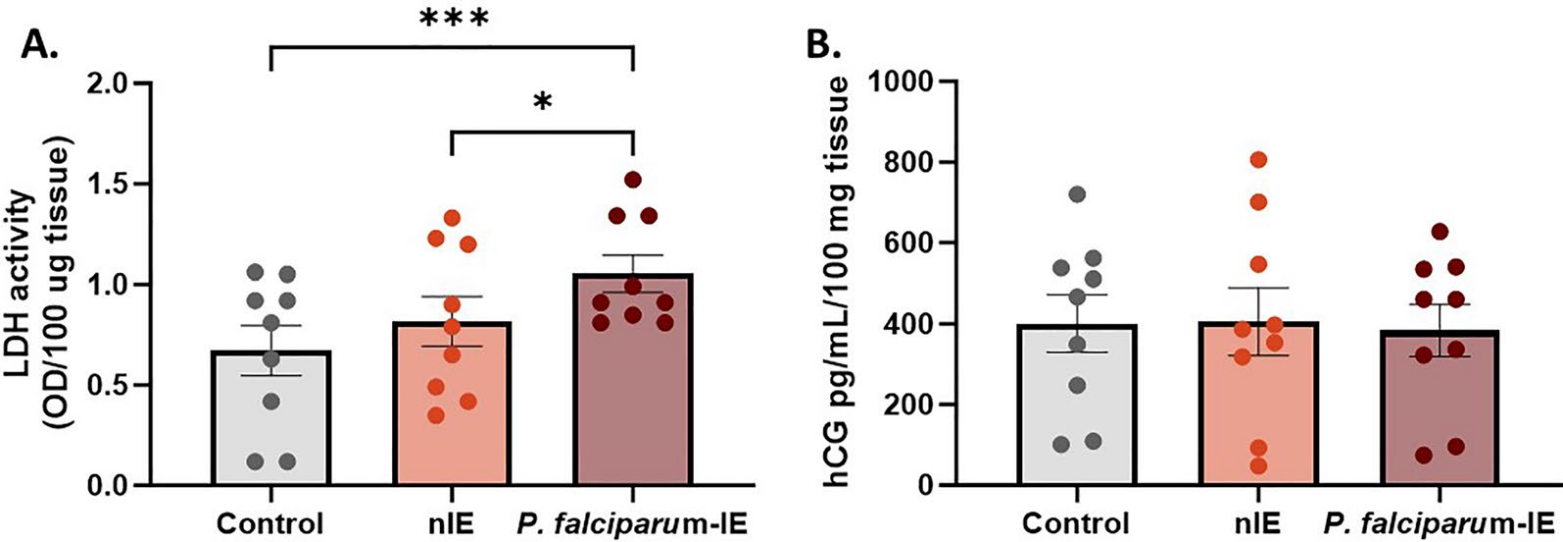
LDH activity and βhCG production in HPEs exposed to *P. falciparum*-IE ex vivo. **A** LDH activity measured in the supernatant of HPEs, normalized per 100 mg of tissue, in the control and treatment groups. **B** Production of βhCG measured in the supernatant of HPEs, normalized per 100 mg of tissue, in the study groups. Bar graphs represent the means ± SEM. Each symbol represents an individual donor (n = 9). One-way ANOVA to repeated measures with a test to multiple comparisons (Tukey)

The main objective of this set of experiments was to evaluate the effect ex vivo of *P. falciparum-*IE on the viability and integrity of placental tissue. The HPEs culture of term placental tissue under conditions that guarantee the viability and structure integrity for up to 72 h of culture was previously adapted [21]. Cell viability was assessed by measuring LDH activity as an indicator of cell damage or lysis. The results obtained from the measurement of this enzyme in HPEs culture supernatants are shown in Fig. 1**.** Placental explants exposed to *P. falciparum-*IE for 24 h showed an increase in cellular cytotoxicity (1.08 ± 0.09) as compared to HPEs exposed to the control using nIEs (0.82 ± 0.12) (*p-value* = 0.0009) (Fig. 1A). A positive control using Triton X-100 that lysates tissue was used as a positive control for the technique, representing the maximum release of LDH into the medium (2.75 ± 0.72). Additionally, the analysis of βhCG secretion in the supernatant of placental explants, which serves as an important biochemical marker of the endocrine activity of syncytiotrophoblast cells within the HPEs, did not reveal significant differences among the treatment groups (*p-value* = 0.845) (Fig. 1B).

### *Plasmodium falciparum-*IE causes histological damage and induces disruption and detachment of trophoblast in HPEs exposed ex vivo

**Fig. 2:**
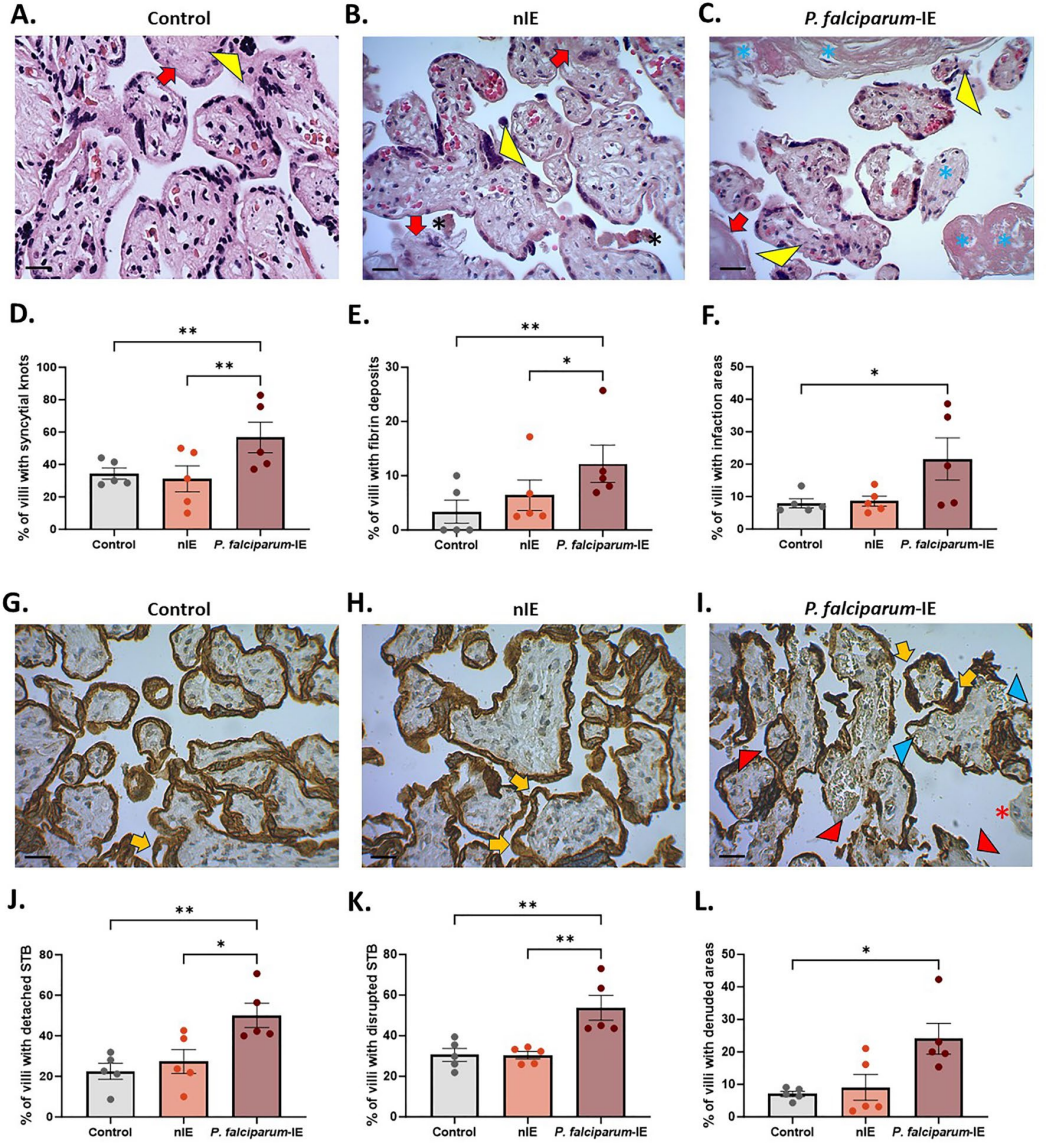
*Plasmodium falciparum-*IE causes histological damage in HPEs exposed ex vivo. **A**–**C** Panel of representative microphotographs of HPEs stained with H&E. **D**–**F** Frequency of the different findings: syncytial knots (yellow arrowhead), fibrin deposits (red arrow), and infarction (blue asterisk). **G**–**I** Panel of representative microphotographs of HPEs stained with CK-7. **J**–**L** Frequency of trophoblast detachment (yellow arrow), trophoblast rupture (red arrowhead), and trophoblast denudation (blue arrowhead). Bar graphs represent the means ± SEM. Each symbol represents an individual donor (n = 5). One-way ANOVA to repeated measures with a test to multiple comparisons (Tukey). Scale bar: 20 μm. Total magnification (×400)

Routine histological analysis was performed using haematoxylin–eosin staining (H&E) to determine the impact of *P. falciparum-*IE on tissue integrity (Fig. 2). Significant increases were observed in the average frequency of syncytial knots (*p-value* = 0.002), fibrin deposits (*p-value* = 0.0003), and infarction (*p-value* = 0.019) in HPEs exposed to *P. falciparum*-IE compared to the control and the HPEs exposed to nIE (Fig. 2D–F). Complementing the H&E analysis, CK-7 immunohistochemistry, a specific marker for trophoblast cells, revealed that *P. falciparum-*IE significantly disrupted the trophoblast of the placental villi (Fig. 2G–I). The frequency of villi presenting detachment of trophoblasts (*p-value* = 0.013), trophoblast rupture (*p-value* = 0.0002), and denudation (*p-value* = 0.003) was significantly higher in the *P. falciparum*-IE group than in the control and nIE groups (Fig. 2J–L).

### *Plasmodium falciparum-*IE disrupts collagen organization in the villous stroma of HPEs exposed ex vivo and induces an increase in areas with thickening of the basal lamina

**Fig. 3:**
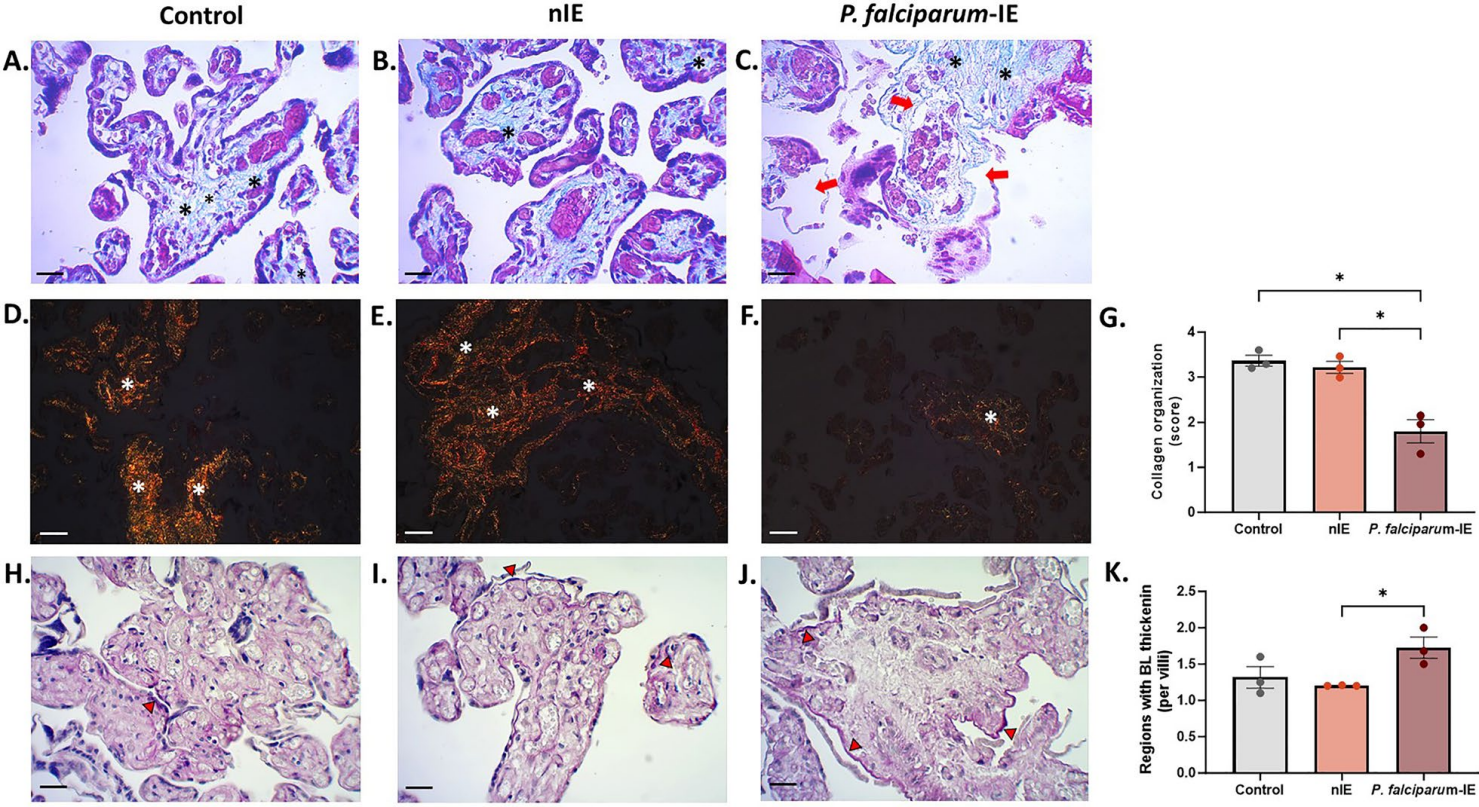
*Plasmodium falciparum-*IE disrupts collagen in the villous stroma of HPEs exposed ex vivo and induces an increase in regions with thickened trophoblast basal lamina. Photographic panel of cross-sections of HPEs stained with: **A**–**C** TM to visualize collagen fiber organization in blue (black asterisk), areas devoid of collagen fibers (red arrows). **D**–**F** PSR (white asterisk). **G** Statistical analysis of the distribution of Col I with PSR. **H**–**J** PAS to identify areas of basal lamina thickening (red arrowhead). **K** Frequency of areas of basal lamina thickening using PAS. Bar graphs represent the means ± SEM. Each symbol represents an individual donor (n = 3). One-way ANOVA to repeated measures with a test to multiple comparisons (Tukey). Total magnification for TM and PAS (×400) (**A**–**C** and **H**–**J)**. Total magnification of PSR (×200) (**D**–**F**). Scale bar: 20 μm

A collagen histochemistry analysis was conducted to assess the integrity of the villous stroma, and the collagen distribution was examined using specific stains. TM staining was performed, facilitating the qualitative identification of collagen fibers (in blue) in the HPEs. Additionally, PRS staining allowed for the specific visualization of collagen I within the villous matrix (in orange) and collagen III within the blood vessels (in green). In the case of TM, large white spaces within the villous stroma indicated the partial or complete absence of collagen in those areas (Fig. 3A–C). Significant differences were noted in both PSR and TM staining sections, indicating severe disorganization of collagen fibers when HPEs were exposed to *P. falciparum*-IE. The distribution of type I collagen by PSR staining (Fig. 3D–F) showed a substantial absence of collagen birefringence in the HPEs group exposed to *P. falciparum*-IE compared to the control samples (*p-value* = 0.011) (Fig. 3G), as indicated by the organization score that decrease from (3.36 ± 0.12) in the control group to (1.80 ± 0.26) in the parasite-treated group.

Another critical component of the villous stroma is the basement membrane, which is part of the placental barrier. PAS reagent is used for the staining regions rich in glycoproteins, typically found in connective tissues and basal lamina [23]; thus, PAS staining was employed to examine this component, with an emphasis on identifying regions with thickened trophoblast basal lamina. In HPEs exposed to *P. falciparum*-IE, an increase in PAS staining was observed (Fig. 3H–J), which was more visible in the basal lamina. Statistical analysis showed a significant increase in the areas of basal lamina with thickening regions per villi in the *P. falciparum*-IE group compared with the nIE group (*p-value* = 0.027) (Fig. 3K).

### *Plasmodium falciparum-*IE does not significantly affect the cellular apoptosis of HPEs exposed ex vivo

**Fig. 4:**
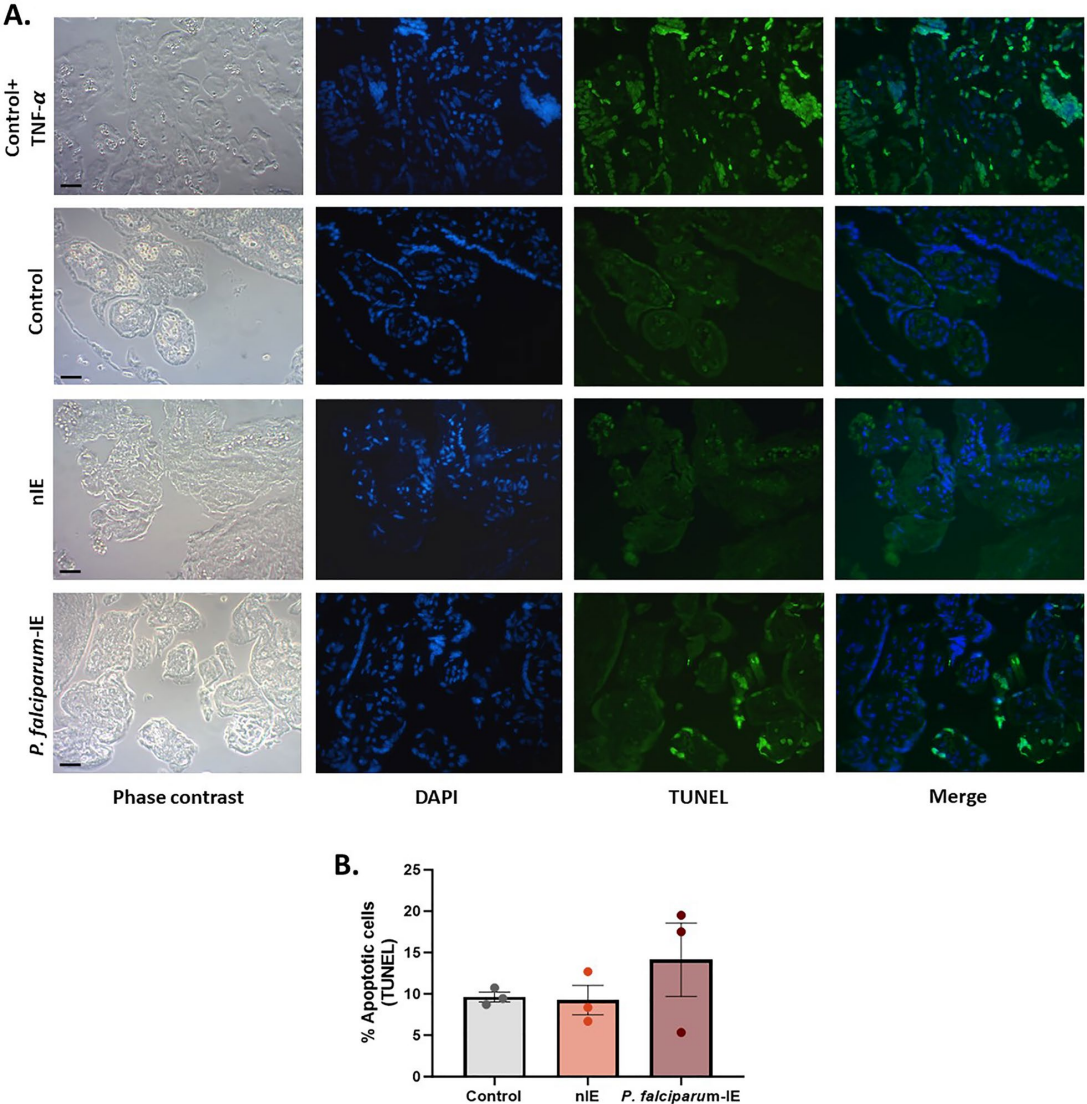
*Plasmodium falciparum-*IE does not significantly affect the cellular apoptosis of HPEs exposed ex vivo. **A** Panel of representative photographs of HPEs exposed ex vivo to *P. falciparum-*IE labelled with TUNEL. The positive control corresponds to HPEs exposed to TNF (20 ng/mL) for 24 h. **B** The frequency of the data is presented in **A**. There are no statistically significant differences in DNA fragmentation among the study groups. Bar graphs represent the means ± SEM. Each symbol represents an individual donor (n = 3). One-way repeated measures ANOVA with multiple comparisons (Tukey) test. Scale bar: 20 μm. Total magnification ×400

Apoptosis in HPEs exposed ex vivo to *P. falciparum-*IE was evaluated using the TUNEL Assay (Fig. 4A). No significant differences were observed among the study groups regarding the percentage of apoptotic cells per villus (*p-value* = 0.37). However, the *P. falciparum*-IE group displayed a positive trend, with 14.13% of apoptotic cells per villus, whereas the Control and nIE groups exhibited very similar percentages of apoptotic cells, around 9% (Fig. 4B).

### The expression of cytokines and angiogenic factors did not change in the placental explants exposed ex vivo to *P. falciparum-*IE

Cytokine and angiogenic factor production were assessed in the study groups to determine whether *P. falciparum-*IE altered these molecules. In general, there were no changes in any molecules, but an increase in IL-6 and IL-10 was observed, although it was not statistically significant (Table 2).

**Table 2:** Production and release of cytokines and angiogenic factors by HPEs exposed ex vivo to *P. falciparum-*IE

Cytokines [pg/mL]	nIE	*P. falciparum*-IE	*p-value*
IL-6	18,887 ± 4658	23,034 ± 6400	0.188
IFN-*γ*	67.6 ± 6.9	76.2 ± 9.5	0.399
IL-4	62.3 ± 7.0	72.9 ± 8.6	0.257
IL-17	37.7 ± 4.2	45.62 ± 5.4	0.228
IL-10	6.1 ± 0.7	9.2 ± 1.8	0.066
IL-2	4.8 ± 0.4	5.67 ± 0.7	0.232
Angiogenic factors [pg/mL]			
sFLT-1	7362 ± 991	7262 ± 1419	0.908
Endoglin	47.8 ± 1.2	55.4 ± 8.1	0.334
PIGF	52.3 ± 3.2	58.2 ± 14.4	0.694
VEGF	44.9 ± 3.8	45.9 ± 4.7	0.841

The cytokine and angiogenic factor production results in HPEs cultured for 24 h with *P. falciparum-*IE and their respective controls. Cytokines (n = 8), angiogenic factors (n = 6). Data represents the means ± SEM, analysed by paired t-test

### Validation of the ex vivo results

#### Collection of placentas naturally exposed to *P. falciparum-*IE infection

To confirm the observed in HPEs exposed ex vivo to *P. falciparum-*IE, placentas of women naturally exposed to infection, hereafter referred to as in vivo exposure to infection, were included. Paraffin-embedded tissues from pregnant women with *P. falciparum* infection were processed as previously described for histological and histochemical analyses with H&E, TM, PSR, PAS, and immunohistochemical analyses with CK-7, as well as for apoptosis assessment using the TUNEL technique. An overview of the characteristics of the pregnant women from whom paraffin-embedded tissues were included is summarized in Table 3. There were no significant differences found between the groups for the characteristics described in the table.

**Table 3:** Characteristics of the pregnant women from the malaria-endemic region of Urabá included in the placental histology analysis

Characteristics	Control n = 6	Past infection n = 6	Active infection n = 6
Maternal age (years)	21.5 ± 3.4	20.3 ± 3.5	25.8 ± 3.6
Multigravid	6 (100%)	6 (100%)	6 (100%)
Previous pregnancies (#)	2.3 ± 1	3.3 ± 2	4.0 ± 1.5
Gestational age (weeks)	40.2 ± 0.7	39.4 ± 2.1	38.3 ± 1.6
Preterm delivery	0	0	0
Placental weight (g)	488 ± 77	490 ± 90	590 ± 86
Birth weight (kg)	3.45 ± 0.24	3.37 ± 0.70	3.41 ± 0.48
Parasites and immune cells in the IVS			
PMNN	14.8 ± 9.0	18.7 ± 9.7	9.8 ± 4.9
Monocytes	12.6 ± 3.2	11.0 ± 5.5	17.8 ± 7.5
Infected erythrocytes (total in 100 HPF)	NA	NA	32.2 ± 49

Parasites and immune cells were counted in 100 fields at 100×. Immune cells are presented as the mean number per field, and parasites are presented as the total of infected erythrocytes counted in the 100 HPF. IVS: Intervillous space, PMNN: Polymorphonuclear neutrophils, HPF: High-power field

**Fig. 5:**
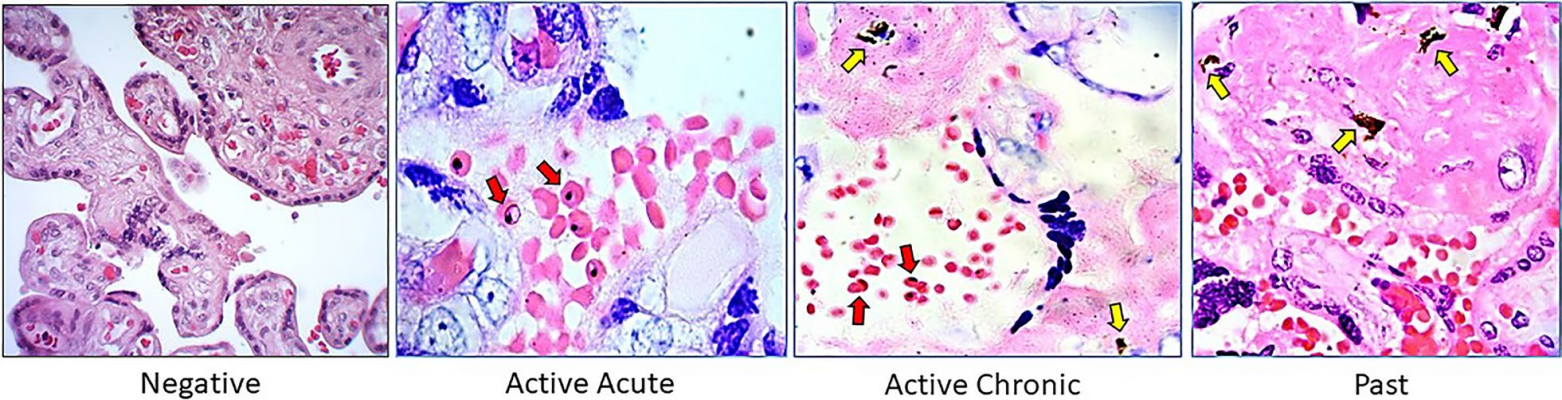
Classification of *P. falciparum* infection in the placenta. *P. falciparum*-IE can be observed in the intervillous space and adhered to the surface of the syncytiotrophoblast (red arrows). Deposits of haemozoin are observed in the fibrin of the villous stroma (yellow arrows). Photos taken by Vásquez AM from the placentas from the Urabá project

For a better understanding of the tissue characteristics included in this study of pregnant women naturally exposed to *P. falciparum-*IE, representative photos are shown in Fig. 5. These tissues were classified as follows: 1. Negative: when no parasitic forms or haemozoin deposits were observed in the tissue or intervillous space, 2. Acute active: when parasitized red blood cells were observed in the intervillous space, 3. Chronic active: when parasitized red blood cells and haemozoin deposits were observed in the tissue, and 4. Past: when only haemozoin deposits are observed in the tissue. Classification by Rogerson et al*.* [24].

### *Plasmodium falciparum-*IE causes histological damage in placental tissue exposed in vivo

**Fig. 6:**
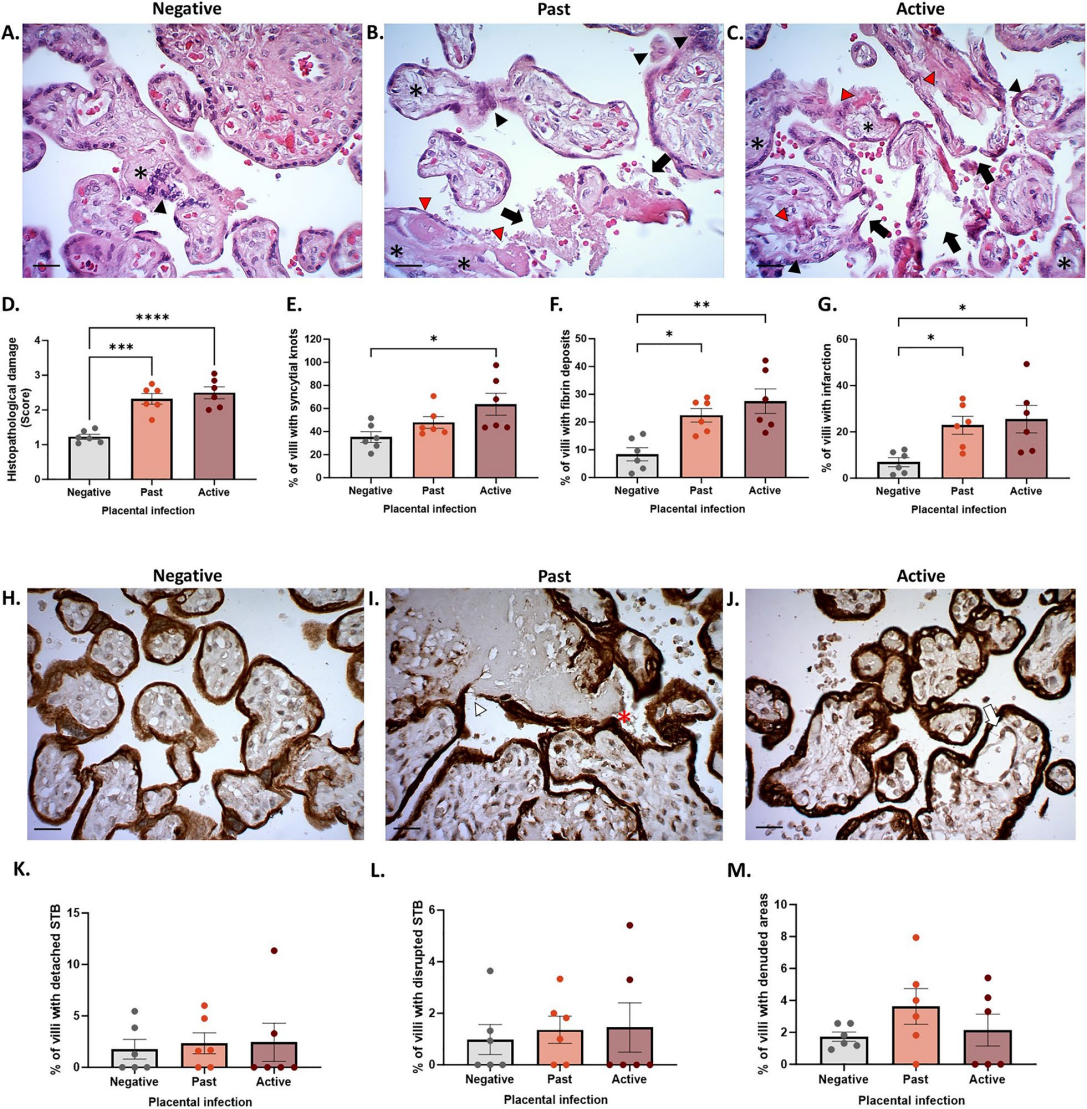
*Plasmodium falciparum-*IE causes histological damage in placental tissue exposed in vivo. **A**–**C** Panel of representative photographs of placental tissue exposed in vivo to *P. falciparum* labelled with H&E. **D**–**G** Statistical analysis panel to determine the frequency of the different findings: histological damage (black arrow), syncytial knots (black arrowhead), fibrin deposits (red arrowhead), and infarction (black asterisk). **H**–**J** Panel of representative photographs of HPEs stained with CK-7. **K**–**M** Frequency of; Trophoblast detachment (white arrow), Trophoblast rupture (white arrowhead), and Trophoblast denudation (red asterisk). Bar graphs represent the means ± SEM. Each symbol represents an individual donor (n = 6). One-way repeated measures ANOVA with multiple comparisons (Tukey) test. Scale bar: 20 μm. Total magnification ×400

Histological analysis with H&E showed a significant increase in histological damage in women with both past and active infection compared to the control group (*p-value* < 0.0001) (Fig. 6D). This damage was reflected in the increased syncytial knots (*p-value* = 0.0306) (Fig. 6E), fibrin deposits (*p-value* = 0.0022) (Fig. 6F), and infarction (*p-value* = 0.1313) (Fig. 6G) which was consistently higher in women with active *P. falciparum* infection in the placenta at the time of delivery. On the other hand, the results obtained with CK-7 staining revealed minimal damage to the trophoblast, with no significant changes observed in terms of detachment, rupture, and denudation between the study groups (Fig. 6K–M).

### *Plasmodium falciparum-*IE disrupts collagen in the stroma of placental tissue exposed in vivo

**Fig. 7:**
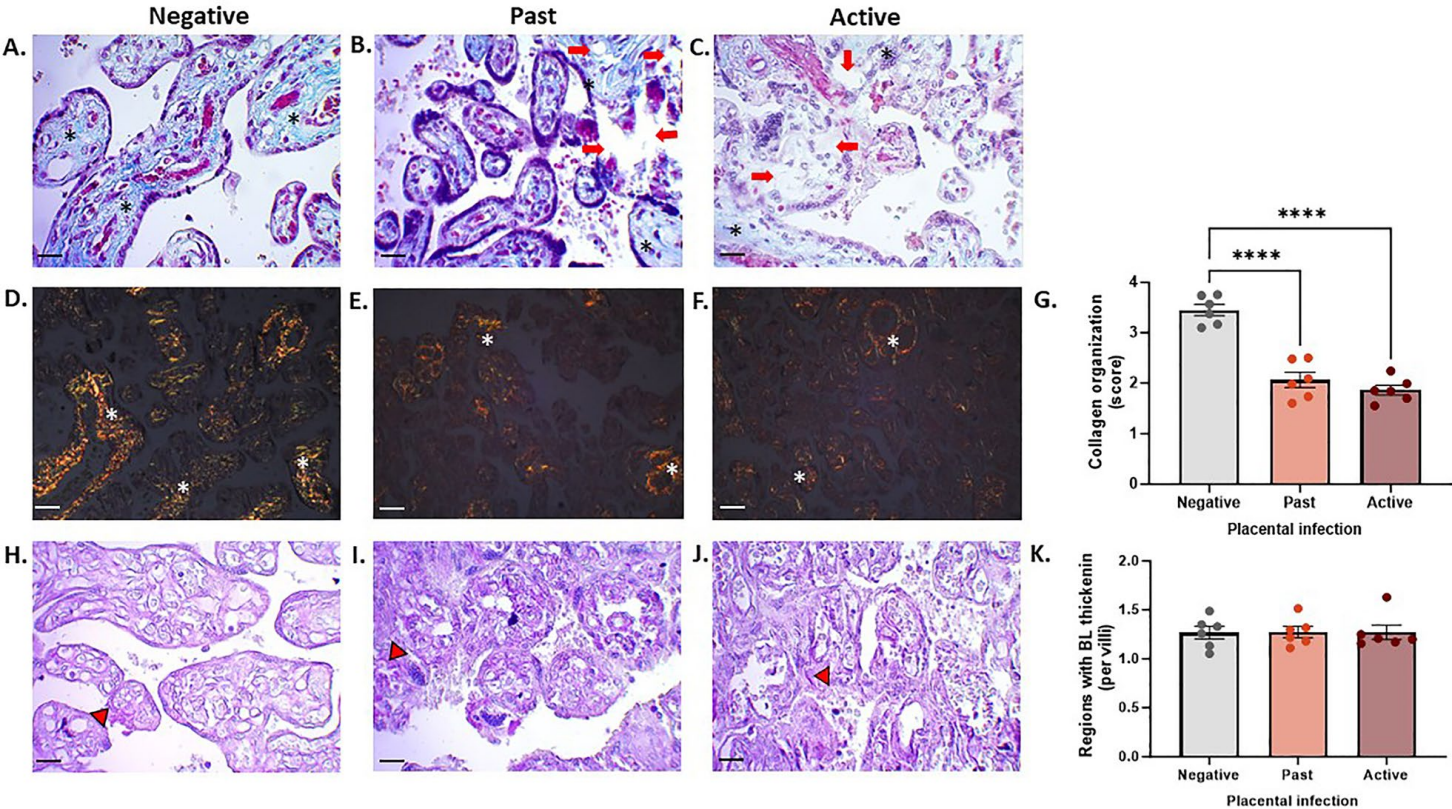
*Plasmodium falciparum-*IE disrupts collagen in the stroma of placental tissue exposed in vivo Photographic panel of cross-sections of placental tissue stained with: **A**–**C** TM to visualize collagen fiber organization in blue (black asterisk), areas devoid of collagen fibers (red arrows). **D**–**F** PSR (white asterisk). **G** Statistical analysis of the distribution of Col I with PSR. **H**–**J** PAS to identify areas of basal lamina thickening (red arrowhead). **K** Frequency of areas of basal lamina thickening with PAS. Bar graphs represent the means ± SEM. Each symbol represents an individual donor (n = 6). One-way ANOVA to repeated measures with multiple comparisons (Tukey). Total magnification for TM and PAS (×400) (**A**–**C** and **H**–**J**). Total magnification for PSR (×200) (**D**–**F**). Scale bar: 20 μm

The qualitative analysis of collagen organization using the TM technique revealed a more significant number of areas in the villous stroma devoid of collagen fibers (Fig. 7A–C), and this could be quantified using the PSR technique (Fig. 7D–F), where an apparent reduction in collagen fibers was more pronounced in the group with both past and active infection than in the control group (*p-value* =  < 0.0001) (Fig. 7G). Regarding the thickening regions of the basement membrane, differences between the study groups were not observed (Fig. 7K).

### *Plasmodium falciparum-*IE significantly affects the cellular apoptosis of placental tissue exposed in vivo

**Fig. 8:**
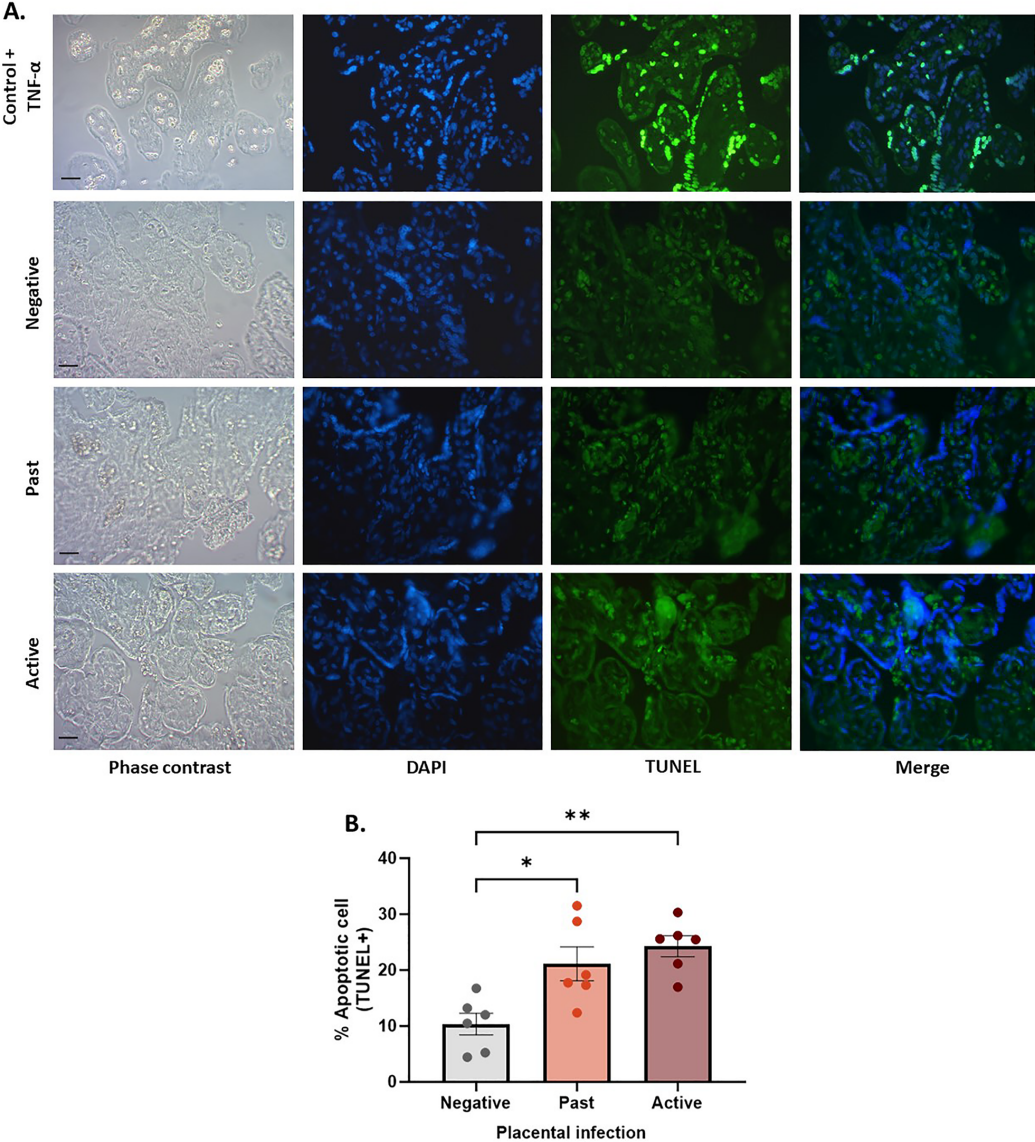
*Plasmodium falciparum-*IE significantly affects the cellular apoptosis of placental tissue exposed in vivo. **A** Panel of representative photographs of placental tissue exposed in vivo to *P. falciparum-*IE labelled with TUNEL. The positive control corresponds to HPEs exposed to TNF (20 ng/mL) for 24 h. **B** The frequency of the data is presented in **A**. Statistically significant differences in DNA fragmentation among the different study groups, with a higher percentage of apoptotic cells in tissues from pregnant women with active and past infection than the control. Bar graphs represent the means ± SEM. Each symbol represents an individual donor (n = 6). One-way repeated measures ANOVA with multiple comparisons (Tukey) test. Scale bar: 20 μm. Total magnification ×400

A significant increase in the percentage of cells undergoing apoptosis was observed in the active and past infection groups compared to the control group (*p-value* = 0.0019). On average, the apoptosis frequency in the active infection group was 24.28% compared to 10.36% in the control group (Fig. 8).

## Discussion

Placental malaria, a severe complication of *P. falciparum* infection during pregnancy, represents a significant public health concern in endemic regions. During infections, *P. falciparum-*IE can accumulate and sequester in the placenta, triggering a cascade of inflammatory responses and tissue destruction. The presence of parasites in the placenta can lead to complications such as maternal anaemia, low birth weight, and intrauterine growth restriction. Additionally, *P. falciparum-*IE can evade the immune system and cause local inflammation, contributing to disruptions in the exchange of nutrients and gases between the mother and the fetus. These disturbances can have a significant impact on fetal development and maternal health.

Despite the greater understanding of placental malaria pathology, the exact mechanisms leading to placental changes and impairment of maternal–fetal exchange are not fully understood. However, it has been suggested that the parasites alone are unlikely to be responsible for placental pathology. Host factors as placental tissue-specific factors and maternal immune response may contribute to the pathological changes. This study aimed to explore the effect of *P. falciparum-*IE on tissue in the HPEs model, and it was found that *P. falciparum-*IE on placental tissue, employing an ex vivo model with human placental chorionic villi explants from third trimester. This model has been widely used to study the impact of congenital parasite protozoan, such as *Trypanosoma cruzi* and *Toxoplasma gondii*. This approach has proven to be a suitable model for studying tissue damage during vertical transmission [16, 17], and provides an opportunity to address unresolved issues related to another significant protozoan parasite that has received limited attention in this model, such as *P. falciparum*.

It was found that *P. falciparum-*IE increases tissue damage in HPEs, disrupting the integrity of the villi, inducing changes in the epithelial barrier and in the villous stroma, which could have implications for the proper functioning of the placental villi, the functional unit of the placenta. This study considered two types of exposure to *P. falciparum*-IE: ex vivo model and in vivo infection, which, in turn, considered different infection times by *P. falciparum-*IE. The short exposure model (ex vivo) involved only a 24-h infection period, whereas the long exposure (in vivo) in the context of naturally exposed pregnant women considered over 24 h of infection and a pre-established immune response by the pregnant woman. Incorporating both models provided a comprehensive understanding, complementing ex vivo results with in vivo findings regarding the effects of *P. falciparum-*IE on placental tissue.

In general, poor placental outcomes associated with histopathological lesions were observed with H&E staining in both ex vivo model and in vivo infection, including infarcts, increased syncytial knots, and fibrin deposits, which have been associated with arterial wall atherosis, accelerated villous maturation, and calcifications in cases of placental malaria [5, 25]. These findings have been reported previously and reflect an association with uteroplacental malperfusion [26] and hypoxia [27]. Fibrin deposits are associated with syncytiotrophoblast damage in placentas affected by placental malaria [28]. Infarction has been associated with poor blood flow perfusion to the fetus and is explained as a pathological change that can contribute to adverse birth outcomes and increased perinatal mortality and morbidity [28, 29]. In HPEs exposed to *P. falciparum-*IE ex vivo*,* evident alterations were found in the integrity of the trophoblast, such as detachment from the stroma and areas of rupture. Collagen disruption in the villous stroma was also observed, leading to increased areas devoid of collagen fibers. These findings are related to the damage observed in LDH release in HPEs supernatant culture, during the exposure to the parasite. In the in vivo infection, no changes were observed in the trophoblast, suggesting that the epithelial layer is constantly replaced, ensuring that there is always trophoblast covering the villi, as previously described [30]. This was not observed in the ex vivo model, possibly due to the short evaluation period, which did not allow sufficient time to replenish the syncytiotrophoblast that detached during infection.

It has been suggested that when there is an alteration in the normal distribution of collagen, changes in the elasticity and strength of placental tissue can occur. These changes may impact the placenta's ability to provide adequate support to the fetus, influence the exchange of nutrients and gases between the mother and the fetus, and compromise the barrier function of the placenta [31, 32]. In the villous stroma, similar observations were made in both the ex vivo model and in vivo infection, with disorganization of collagen fibers. This suggests that even if the trophoblast is replenished in vivo, stromal damage persists in the tissue, or the complete recovery is slower [33].

Regions with thickening of the trophoblast basement membrane increased in the exposed HPEs compared to the unexposed group, which is related to previous findings in naturally exposed placentas [34]. The thickening of the basement membrane can be seen from two angles: one from the perspective of a consequence of the infection and a possible pathogenic mechanism that explains the decrease in nutrient and oxygen transport through the placental barrier, and second, from the host's perspective, where it could be hypothesized as a possible defense mechanism that prevents infection in regions without trophoblast [35]. Interestingly, it has been suggested that the production of non-chemotactic cytokines may be associated with the thickening of the trophoblast basement membrane and may cause a mechanical blockage in the transport of oxygen and nutrients in the placenta [36].

To elucidate the discrepancy in the increased LDH levels without a corresponding increase in apoptosis detected by the TUNEL technique, we may consider that the elevated LDH could indicate damage to the cell membrane, leading to LDH release from cells. This could be associated with cellular damage from lysis and necrosis occurring in the HPE model exposed to *P. falciparum*-IE for 24 h, an exposure that did not necessarily result in apoptosis. It is plausible that longer exposure periods could induce apoptotic processes.

Apoptosis, which did not show significant differences in the ex vivo model, showed a significant increase in placentas from pregnant women with an active infection by *P. falciparum-*IE*,* where the exposure to parasites was more prolonged*.* This suggests that more time may be needed to demonstrate the effect of apoptosis ex vivo due to the complex process involving various factors [37]. It is necessary to mention that the increase in apoptosis may also be related to the formation of syncytial knots and constant trophoblast regeneration [30, 38]. A limitation of this study was the absence of caspase evaluation, for example, caspase 8, which hinders the verification of the cellular apoptosis process [39].

The production of cytokines and angiogenic factors were only evaluated in the ex vivo infection model. In this model, the specific production of cytokines in villous cells could be studied because maternal blood was removed before culturing and examining the products secreted by the villi into the medium A trend toward an increase in all evaluated cytokines was observed in the *P. falciparum*-IE group, which aligns with previous reports [40, 41], especially the significant increase in IL-6. This may be related to the imbalance in the immune response induced by *P. falciparum* infection in the human placenta [42]. However, angiogenic factors did not show changes between the evaluated groups, which may be due to the exposure time, which may need to be longer to generate an effect related to angiogenic function, as has been observed in placentas naturally exposed, with a significant increase in anti-angiogenic factors such as sFLT-1 [43, 44].

In general, taken together, the results obtained allow the identification of extensive disruption of placental histology and function, which may result in fetal loss or impaired intrauterine growth [45]. Which are clinical symptoms that can be recognized in cases of congenital malaria [46]. The frequency of cells undergoing apoptosis has been reported to be significantly higher in the presence of congenital infection, even in asymptomatic and submicroscopic infections [47]. This cell death could partly explain the intrauterine growth restriction as previously described [48] and in other infectious processes the cell death could be increased such as cytomegalovirus [49] and *Toxoplasma gondii* [50] and *Trypanosoma cruzi* [23].

The HPEs model offers the advantage of maintaining intact microarchitecture and preserving cell–cell interactions and paracrine communications. Therefore, the contribution of mesenchymal and endothelial cells in metabolic processes and their effects on tissue integrity should be considered [51, 52]. Interestingly, findings reflected characteristics specific to each model, mainly the exposure time to infection and the participation of extracellular matrix components and other factors present in the in vivo environment versus ex vivo as mechanisms of tissue repair or maternal immune response to damage. These are factors that possibly allowed some findings to be different [53].

## Limitations of the study

There is a lack of a model to study *P. falciparum-*IE interaction with the human placenta in the context of pregnancy malaria. The experimental approach presented here allowed us to evaluate the impact of malaria parasites on the placental integrity and function, providing information regarding the first interaction between the placenta and the parasite, in a short period of time. However, some important limitations should be mentioned.

The negative effects observed on the tissue cannot exclusively be attributed to the direct contact of infected erythrocytes with the tissue. HPEs were exposed to late trophozoites and schizonts, and during parasite development, mature schizonts rupture and release several parasite-derived products such as haemozoin, glycosylphosphatidylinositol (GPI), parasite proteins, and DNA, among others, that can modulate the response of placenta cells. Future studies aim to elucidate the specific role of cytoadherence can control these factors, for example, by removing adherence proteins from the erythrocyte surface with trypsin, using *P. falciparum* strains that do not adhere to CSA, shortening incubation times, and testing the effect of soluble parasite factors released during the *Plasmodium* replication cycle.

Although the HPEs model was helpful in evaluating the initial response of the human placenta to the interaction with infected erythrocytes, it is not an appropriate model for the assessment of the long-term outcomes influenced by maternal immune components. Due to the nature of this model, involving maternal blood removal by washing the tissue before the culture of explants, the maternal immune component was removed. Consequently, an exaggerated immune response was not observed in the placental explants exposed to the infected erythrocytes, and the little response observed may be primarily mediated by the villous cellular component, particularly Hofbauer cells. While this study does not comprehensively replicate the entire in vivo scenario, its aim is to illustrate the direct interaction of *P. falciparum-*IE with placental villi without maternal response intervention.

Another limitation is related to the parasite exposure time, that in this study was over a 24-h period. Future studies should consider including other time points, to gain clarity on how variable the effect of plasmodial infection can be at different time points, for example since the beginning of the parasite-tissue interaction, or even latter simulating more prolonged exposition, that allows to explore tissue damage repair mechanisms activated.

Finally, since third-trimester placentas were used, conclusions regarding the impact of parasite exposure during early pregnancy cannot be drawn.

In summary, the results obtained allow us to identify that the mere presence of the parasite already exerts a negative impact on the tissue. Whether through adherence mechanisms or the parasite's life cycle, during which numerous factors may be released, both cytoadherence and soluble products interact with villous cells, subsequently activating cascades of inflammatory processes that may contribute to resolving the infection or exacerbating inflammation and tissue damage. On the other hand, the interaction of parasitized erythrocytes with tissue cells may promote repair, regeneration, or cytotoxicity mechanisms, which can be evidenced in trophoblast remodelling or increased activity of factors such as LDH.

## Conclusions

*Plasmodium falciparum-*IE induces damage to the trophoblast in the ex vivo infection model, which was not observed in the in vivo infection. This discrepancy may be attributed to the limited exposure time in the ex vivo model, preventing the syncytial regeneration seen in the in vivo tissue. The alteration in the villous stroma was a common finding in both exposure models, and it is of particular significance as it could indicate irreversible damage that persists regardless of the duration of exposure. This alteration may serve as a mechanism that disrupts placental barrier function. The basement membrane, another critical component of the placental barrier, was affected in the ex vivo model but remained intact in the in vivo infection. This suggests that it might serve as a defense mechanism when the trophoblast is detached, damaged, or exposed. Further studies exploring the impact of *P. falciparum-*IE could provide additional insights to complement the findings presented here. Incorporating additional components in the functional assessment of tissues, such as protein transport, may help uncover other specific mechanisms underlying impaired tissue function. The findings of this study could have implications for future management guidelines in regions endemic to malaria.

### Supplementary Information

**Additional file 1. **General characteristics of placental donor and sample size by experiment.

**Additional file 2. **Summary of placental malaria study findings.

## Data Availability

The authors confirm that the data supporting the findings of this study are available within the article.
