# Supplementary material for: Plasmodium falciparum alters the trophoblastic barrier and stroma villi organization of human placental villi explants
Source: Malar J. 2024 May 1;23:130. doi: 10.1186/s12936-024-04960-9 (PMC11064279; doi:10.1186/s12936-024-04960-9)
Supplement: Supplementary file 1 — Additional file 1. General characteristics of placental donor and sample size by experiment. [file 12936_2024_4960_MOESM1_ESM.docx]

**Additional file 1. General characteristics of placental doner and sample size by experiment.**

| **Placenta donor** | **Project code** | **Age** | **Gestational age** | **Newborn sex** | **LDH and hCG** | | | **Histological analysis-HE** | | | **Immunostaining (CK-7)** | | | **Collagen and PAS staining** | | | **TUNEL** | | | **Cytokines** | | **Angiogenic factors** | |
| --- | --- | --- | --- | --- | --- | --- | --- | --- | --- | --- | --- | --- | --- | --- | --- | --- | --- | --- | --- | --- | --- | --- | --- |
|  |  |  |  |  | C- | nIE | Pf-IE | C- | nIE | Pf-IE | C- | nIE | Pf-IE | C- | nIE | Pf-IE | C- | nIE | Pf-IE | nIE | Pf-IE | nIE | Pf-IE |
| 1 | P10 | 40 | 39 | Female | ✓ | ✓ | ✓ |  |  |  |  |  |  |  |  |  |  |  |  | ✓ | ✓ | ✓ | ✓ |
| 2 | P12 | 33 | 38+5 | Female | ✓ | ✓ | ✓ | ✓ | ✓ | ✓ | ✓ | ✓ | ✓ | ✓ | ✓ | ✓ | ✓ | ✓ | ✓ | ✓ | ✓ | ✓ | ✓ |
| 3 | P13 | 35 | 38 | Male | ✓ | ✓ | ✓ | ✓ | ✓ | ✓ | ✓ | ✓ | ✓ | ✓ | ✓ | ✓ | ✓ | ✓ | ✓ | ✓ | ✓ |  |  |
| 4 | P14 | ND | 39 | Female | ✓ | ✓ | ✓ | ✓ | ✓ | ✓ | ✓ | ✓ | ✓ | ✓ | ✓ | ✓ | ✓ | ✓ | ✓ | ✓ | ✓ |  |  |
| 5 | P16 | 34 | 38 | Female | ✓ | ✓ | ✓ | ✓ | ✓ | ✓ | ✓ | ✓ | ✓ |  |  |  |  |  |  | ✓ | ✓ | ✓ | ✓ |
| 6 | P17 | 33 | 39 | Male | ✓ | ✓ | ✓ | ✓ | ✓ | ✓ | ✓ | ✓ | ✓ |  |  |  |  |  |  | ✓ | ✓ |  |  |
| 7 | P18 | 33 | 38+1 | Male | ✓ | ✓ | ✓ |  |  |  |  |  |  |  |  |  |  |  |  | ✓ | ✓ | ✓ | ✓ |
| 8 | P20 | 32 | 39 | Female | ✓ | ✓ | ✓ |  | ✓ | ✓ |  | ✓ | ✓ |  |  |  |  |  |  | ✓ | ✓ | ✓ | ✓ |
| 9 | P21 | 32 | 39+3 | Male | ✓ | ✓ | ✓ |  | ✓ | ✓ |  | ✓ | ✓ |  |  |  |  |  |  | ✓ | ✓ | ✓ | ✓ |
| **Sample size (donor) by experiment** | | | | | **9** | | | **5** | | | **5** | | | **3** | | | **3** | | | **9** | | **6** | |

C-: negative control (HPE in culture media); nIE: non-infected erythrocytes; Pf-IE: infected erythrocytes by *P. falciparum.*
