# Supplementary material for: Plasmodium falciparum alters the trophoblastic barrier and stroma villi organization of human placental villi explants
Source: Malar J. 2024 May 1;23:130. doi: 10.1186/s12936-024-04960-9 (PMC11064279; doi:10.1186/s12936-024-04960-9)
Supplement: Supplementary file 2 — Additional file 2. Summary of placental malaria study findings. [file 12936_2024_4960_MOESM2_ESM.docx]

**Additional file 2. Summary of Placental Malaria Study Findings**

| **Aspect/Model** | ***Ex vivo* Model** | ***In vivo* infection** |
| --- | --- | --- |
| Exposure Time | 24 hours | Over 24 hours |
| Histopathological Lesions | Infarcts, syncytial knots, fibrin deposits. | Similar findings as *ex vivo* model. |
| Trophoblast Integrity | Altered, detachment from stroma, areas of rupture. | No observed changes, constant replenishment. |
| Collagen Disruption | Evident in villous stroma. | Persistent in villous stroma. |
| Basement Membrane Thickness | Increased in exposed HPEs. | Related to active infection in naturally exposed placentas. |
| Apoptosis | No significant differences in *ex vivo* model. | Significantly increased in placentas with active infection. |
| Cytokine Production | Trend towards increase, especially IL-6 | Not evaluated in *in vivo* infection. |
| Angiogenic Factors | No significant changes. | Not evaluated in *in vivo* infection. |

Clear breakdown of findings from both the *ex vivo* model and *in vivo* infection, allowing for easy comparison across different aspects of the study.
